# Use of Emergency Manuals to Treat Delayed Emergence After Robotic-Assisted Cholecystectomy

**DOI:** 10.7759/cureus.10660

**Published:** 2020-09-26

**Authors:** Wayne R Simmons, Pavit S Deol, Ali Ahmed-Elamin, Jeffrey Huang

**Affiliations:** 1 Anesthesiology, HCA Healthcare/University of South Florida (USF) Morsani College of Medicine Graduate Medical Education (GME) Programs: Oak Hill Hospital, Brooksville, USA; 2 Anesthesiology, University of South Florida Morsani College of Medicine, Tampa, USA

**Keywords:** anesthesiology, delayed emergence, emergency manual, cognitive aid

## Abstract

Delayed emergence is defined as failure to regain consciousness 30 to 60 minutes after general anesthesia. Although incidence is low and difficult to accurately estimate, there is a wide differential diagnosis to be considered. Emergency manuals (EMs) are visual cognitive aids that can be used in the operating room to help manage intraoperative complications. They provide immediate access to evidence-based guidelines to optimize management of intraoperative complications. They are being increasingly implemented in the clinical setting and have been shown to improve patient safety. A case of a patient with delayed emergence after undergoing robotic-assisted cholecystectomy is described here. The delayed emergence section of the Stanford Anesthesia Emergency Model was referenced immediately and guided management of the patient. Utilization of an EM resulted in rapid return to baseline mental status. EMs allow health care providers to respond to intraoperative scenarios efficiently and effectively and ultimately improve patient care.

## Introduction

Delayed emergence can be defined as failure to regain consciousness 30 to 60 minutes after general anesthesia [1]. The clinical presentation can often involve both altered mental status and respiratory complications. The low incidence of delayed emergence has limited research. However, one study involving over 13,000 surgical patients in New Zealand found that prolonged sedation occurred at a rate of 0.25%, or 1 in 400 patients [2]. Most common causes of delayed emergence include residual anesthetic agents, drug interactions and polypharmacy [3]. Other possible diagnoses include surgical complications, neurologic sequelae, endocrine disturbance, metabolic derangement and psychiatric conditions [1-4].

Given the difficulty in determining the true incidence of delayed emergence and its multiple causes, prior preparation and readiness are vital when these situations arise. Studies have shown that humans in general do not optimally retrieve details from long-term memory during stressful situations, but operating room teams deliver best practices more often and efficiently when using cognitive aids [5-8]. Operating room emergency manuals (EMs) contain cognitive aids with evidence-based guidelines on the management of specific intraoperative critical events [7]. EMs also increase immediate access to resources and decrease reliance on rote memory alone [5]. The use of operating room EMs has been shown to allow anesthesia professionals to respond more confidently and accurately to critical events [5-9].

## Case presentation

A 27-year-old Caucasian female (weight, 53.6 kg; height, 154.95 cm; body mass index, 22.3 kg/m^2^; American Society of Anesthesiology physical status II) was scheduled for a robotic-assisted cholecystectomy. Her past medical history included gastroesophageal reflux, anxiety, depression and temporomandibular joint dysfunction. Her home medications included prescribed hydrocodone bitartrate/acetaminophen 5-325 mg tablets as needed for acute abdominal pain and testosterone cypionate 200 mg/mL as a 0.25 mL injection every week for transgender hormone therapy. The patient had allergies to cefaclor and diphenhydramine.

On exam she was awake, alert and orientated with preoperative laboratory values within normal limits. Blood pressure was measured to be 110/84 with sinus rhythm at a heart rate of 60. The patient was given 2 mg midazolam in the preop area and afterwards was brought to the operating room. Her monitoring included electrocardiography, noninvasive blood pressure, pulse oximetry, capnography and temperature measurements. After preoxygenation, anesthesia was induced in the supine position with 2 mg/kg propofol, 200 mcg fentanyl, and 0.6 mg/kg rocuronium. After intubation, anesthesia was maintained with sevoflurane in 100% oxygen. During the operation, the patient received 1,000 mL of lactated ringers’ solution and remained hemodynamically stable throughout. The only dose of fentanyl was given during induction. An additional 10 mg of muscle relaxant was administered 15 minutes before the end of the surgery. The entire procedure took 30 minutes to complete, and the blood loss was minimal at about 15 mL. The neuromuscular blockade was reversed with 0.07 mg/kg neostigmine and 0.8 mg of glycopyrrolate. After emergence she was breathing spontaneously with stable vital signs, but was maintaining inadequate tidal volumes without proper response to verbal commands or painful stimuli.

**Figure 1:**
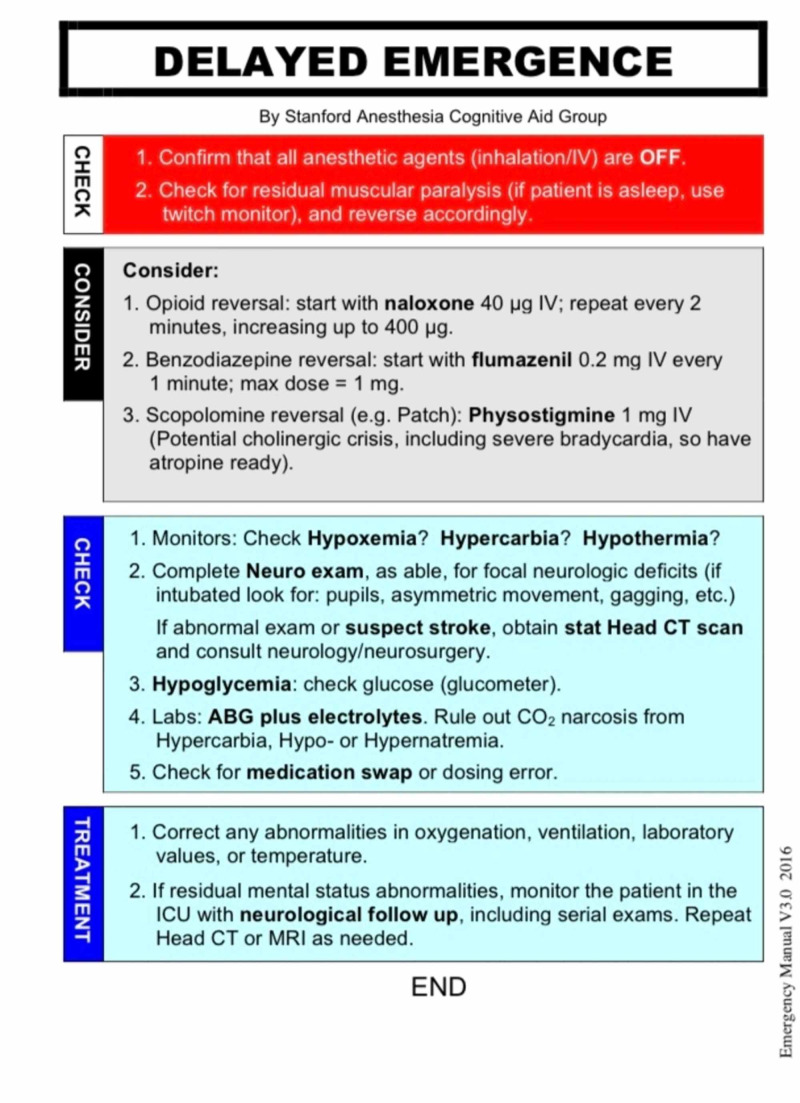
Delayed Awakening section of the Stanford Emergency Manual V3.0 2016 Emergency Manual [10]. Permissions provided under creative commons license without alteration or use for commercial purposes [11].

To provide optimal care the attending anesthesiologist and resident used the Stanford Anesthesia Emergency Manual checklist (Figure 1) on the management of delayed emergence to verify proper treatment. The inhalation agent had been previously discontinued, and the sevoflurane level was near zero on the monitor. Her temperature was 35.80 C, and oxygen saturation was 99% with pink lip color. Her pupils were constricted but were not pinpoint. End-tidal CO2 (EtCO2) was 45 mmHg with inadequate spontaneous breathing and train-of-four (TOF) monitoring showed 4/4 twitches. Sugammadex 200 mg was given intravenously (IV) to ensure rocuronium reversal, however, the patient remained unresponsive to verbal commands or painful stimuli. Naloxone 0.2 mg IV was also given for opioid reversal, after which the patient began to respond to verbal commands and maintain tidal volumes of roughly 7 mL/kg. She was extubated in the operating room and was transferred to the post-anesthesia care unit (PACU). Postoperative vital signs were within normal limits and the remaining post-operative course was uneventful with no post-operative complications.

## Discussion

Delayed emergence in anesthesia is relatively uncommon and has a wide differential diagnosis including life threatening neurologic or endocrine disturbances, making it important to consider the various causes when dealing with this complication. After confirming that all anesthetic agents are discontinued and checking for residual muscular paralysis, various regimens can be given for reversible causes [10]. Naloxone can be given for opioid reversal, flumazenil for benzodiazepine reversal, and physostigmine for scopolamine reversal [10]. Monitors and labs may be checked for hypoxemia, hypercarbia, hypothermia, hypoglycemia and electrolyte abnormalities, and any abnormalities should be corrected [10]. The entire clinical picture must be considered when evaluating for potential causes since factors such as patient comorbidities and polypharmacy may help guide treatment.

This patient was 27 years old and otherwise healthy. Her age and comorbidities were not significant factors and were unlikely to contribute to delayed emergence. The types of anesthetic drugs administered for the procedure were the main concern. Opioids can cause respiratory depression, hypercarbia and sedation, which can delay emergence from general anesthesia. Therefore, naloxone, a μ receptor antagonist, was used in this case. The patient had been using opioid for pain relief before surgery. Therefore, more than 2 ug/ml fentanyl was administered during induction. She did not have pinpoint pupils. Furthermore, a peripheral nerve stimulator can help make the diagnosis of incomplete reversal of nondepolarizing muscle relaxants that result in paralysis. This patient had 4/4 twitches and resumed spontaneous breathing with small tidal volumes after initial neuromuscular blocking reversal agents were given. Additionally, sugammadex was given without noticeable effect in order to rule out incomplete reversal of nondepolarizing muscle relaxants. Additional causes of inadequate tidal volumes during emergence from anesthesia can be agitation and distress caused by pain, as well as central nervous system depression from excessive benzodiazepine administration. This patient received 2 mg of midazolam nearly 30 minutes prior to the procedure, and it was assumed that any effect on delayed emergence was unlikely. Therefore, the benzodiazepine reversal agent flumazenil was not administered.

Since studies have shown that humans lack optimal recall of long-term memory during stressful situations, cognitive aids such as EMs can be used to ensure that best practices are consistently being delivered [5-8]. Although EMs have demonstrated benefit in simulation and clinical settings, it is important to train clinicians in the use of the EMs on content, format and location [6,12]. At our institution, a hard copy of the EM is located at each anesthesia station and trainees are encouraged to have digital copies on their mobile devices for immediate availability. In this case, the Stanford Anesthesia EM allowed rapid verification of pertinent monitoring and treatments [12]. The administration of naloxone in a timely manner ultimately allowed the patient to quickly return to baseline mental status and transition care to the PACU in stable condition. Similarly, use of EMs by anesthesiologists has also been shown in previous reports to successfully respond to intraoperative supraventricular tachycardia, hypotension, hemorrhage, hypoxemia, and bradycardia [13-15].

## Conclusions

The effective application of an EM to the management of delayed emergence resulted in a return to baseline mental status without further complications. This case adds to the growing body of evidence that EMs provide an effective and efficient way to improve patient safety by providing immediate evidence-based medical guidelines for management of intraoperative complications where pure reliance on memory may not be optimal. EMs are being implemented worldwide and have been shown to improve patient safety and harbor a culture of teamwork. In the case presented here, the use of EM improved patient care and provided an immediate reference in managing delayed emergence. Further research on the use of EMs should be conducted to continue to evaluate their efficacy in the operating room setting.
